# *Thorsmoerkia curvula* gen. et spec. nov. (Trebouxiophyceae, Chlorophyta), a semi-terrestrial microalga from Iceland exhibits high levels of unsaturated fatty acids

**DOI:** 10.1007/s10811-021-02577-y

**Published:** 2021-09-09

**Authors:** Cecilia Nicoletti, Lenka Procházková, Linda Nedbalová, Réka Mócsai, Friedrich Altmann, Andreas Holzinger, Daniel Remias

**Affiliations:** 1 School of Engineering, University of Applied Sciences Upper Austria, Stelzhamerstr. 23, 4600 Wels, Austria; 2 Department of Ecology, Faculty of Science, Charles University, Viničná 7, 12844 Prague, Czech Republic; 3 Centre for Phycology, Institute of Botany of the Czech Academy of Sciences, Dukelská 135, 37982 Třeboň, Czech Republic; 4 Department of Chemistry, University of Natural Resources and Life Sciences, Muthgasse 19, 1190 Vienna, Austria; 5 Department of Botany, University of Innsbruck, Sternwartestraße 15, 6020 Innsbruck, Austria

**Keywords:** Biodiversity, Iceland, Polyunsaturated fatty acids, Semi-terrestrial algae, Trebouxiophyceae, Taxonomy

## Abstract

A terrestrial green alga was isolated at Iceland, and the strain (SAG 2627) was described for its morphology and phylogenetic position and tested for biotechnological capabilities. Cells had a distinctly curved, crescent shape with conical poles and a single parietal chloroplast. Phylogenetic analyses of 18S rDNA and *rbc*L markers placed the strain into the Trebouxiophyceae (Chlorophyta). The alga turned out to belong to an independent lineage without an obvious sister group within the Trebouxiophyceae. Based on morphological and phylogenetic data, the strain was described as a new genus and species, *Thorsmoerkia curvula* gen. et sp. nov. Biomass was generated in column reactors and subsequently screened for promising metabolites. Growth was optimized by pH-regulated, episodic CO_2_ supplement during the logarithmic growth-phase, and half of the biomass was thereafter exposed to nitrogen and phosphate depletion. The biomass yield reached up to 53.5 mg L^−1^ day^−1^. Fatty acid (FA) production peaked at 24 mg L^−1^ day^−1^ and up to 83% of all FAs were unsaturated. At the end of the log phase, approximately 45% of dry mass were lipids, including eicosapentaenoic acid. Carotenoid production reached up to 2.94 mg L^−1^ day^−1^ but it was halted during the stress phase. The N-linked glycans of glycoproteins were assessed to reveal chemotaxonomic patterns. The study demonstrated that new microalgae can be found at Iceland, potentially suitable for applied purposes. The advantage of *T. curvula* is its robustness and that significant amounts of lipids are already accumulated during log phase, making a subsequent stress exposure dispensable.

## Introduction

Microalgae living in ecosystems with harsh abiotic conditions are of general interest in terms of biodiversity, physiology, or biotechnology. Valuable cellular compounds like pigments or polyunsaturated fatty acids (PUFAs) can be abundantly present (Paliwal et al. 2017). On Iceland, extreme climatic conditions occur in vast areas of the back-country, where open soils, rocks, or snow and ice surfaces prevail (Arnalds 2015). Nonetheless, the island has hardly been surveyed for terrestrial microbial phototrophs, and the few studies focused on biodiversity (Broady 1978; Furey et al. 2020) or limited to specialized communities living on glaciers and ice caps. The latter communities were characterized by Lutz et al. (2015), and secondary carotenoids like astaxanthin were observed in this cryoflora. However, cultivation of such pigment-rich cysts remains an obstacle, hindering applied use of snow or glacial algae so far (Hoham and Remias 2020).

The exploration of microalgae adapted to the climatic conditions of Northern countries is advantageous particularly for the recruitment of candidates for local biomass production including high-value compounds (Spijkerman et al. 2012; Leya 2020), or for water remediation performed at lower temperatures (Cheregi et al. 2019). Generally, these organisms are a well-known and rich source of polyunsaturated fatty acids (Kumar et al. 2019), and in the last years, further compounds like polyphenols, carotenoids, vitamins, sterols, or mycosporine-like amino acids (MAAs) came into biotechnological focus (Sansone and Brunet 2019). In the case of Trebouxiophyceae, Mócsai et al. (2020b) recently showed that characteristic N-glycans were present and these can possibly support the taxonomic identification.

For this study a green microalga isolated from a diverse semi-terrestrial community on wet gravel in southern Iceland was selected. It exhibited a characteristic morphology, turned out to be growing well in bioreactors, exhibited high levels of unsaturated fatty acids already during the growth phase, and showed excellent handling during harvest and analytical post-processing, thus possessing biotechnological capabilities. In accordance with the guideline of Fawley and Fawley (2020) for the identification of novel microalgal strains, morphologic and phylogenetic analyses were conducted to find the closest relatives. As this strain has an uncertain phylogenetic position, we suggested a new taxon and discuss its placement within the Trebouxiophyceae. This diverse class is currently undergoing a revision; several genera are found to be polyphyletic (e.g., Dal Grande et al. 2014) and many new species and genera have been described (e.g., Darienko and Pröschold 2019; Mikhailyuk et al. 2020).

## Material and methods

### Field collection and cultivation

On 11 July 2017, macroscopic algal mats were harvested from wet volcanic gravel at N63°42.144, W19°40.194, 157 m a.s.l. in at Þórsmörk, Iceland (sample WP156). For photoautotrophic species isolation, 50 μL of the suspended community was inoculated on sterile agar plates (1.7% w/v agar) on “Synthetic Freshwater Medium” (SFM, composition accessible at https://www.uni-due.de/biology/ccac/growth_media_sfm.php) at pH 6.2, 15 °C, and 25 to 40 μmol photons m^−2^ s^−1^ (14-h light/10-h darkness). Green colonies were picked from the plates after several weeks and re-inoculated on fresh plates until a unialgal strain, evaluated by light microscopy (LM), was gained. The alga was deposited at the SAG strain collection (Göttingen, Germany) as SAG 2627. Prior further use in the lab, cells were transferred from petri dishes into 250-mL Erlenmeyer flasks with liquid SFM, shaken at 80 rpm.

### Experimental design

Open-top glass column reactors with a capacity of 1.2 L were used at 20 °C in triplicate for biomass generation at 1000 or 1140 mL working volume. In contrast to stock cultures in Erlenmeyer flasks, the pH was changed from 6 to 7 and SFM was modified containing twice nitrogen and phosphate for increasing growth rates (“eSFM”, receipt see Supplemental Table 1). Irradiation was provided 14 h day^−1^ with Narva LT18W/958 Bio Vital fluorescence tubes (Lumiled, Aachen, Germany) at approximately 100 to 120 μmol photons m^−2^ s^−1^. Continuous air flow through the liquid culture was provided from the bottom with sterile-filtered compressed air at flow rate of 1 L min^−1^. Two assays were performed, each with growth and stress phase: (a) conventional aeration, (b) aeration and with CO_2_ supplementation, and finally (c & d) the same setups, but with stress medium (SFM lacking vitamins, nitrogen, and phosphate; details see Supplemental Table 1). The episodic CO_2_ supplementation was provided as follows (for “growth + CO_2_,” “stress + CO_2_”): once pH exceeded 7.2 (due to photosynthetic HCO_3−_ uptake), a magnetic valve was opened automatically to nourish the air with CO_2_ and thus to instantly decrease the pH value in the reactor to approximately 5.5 (JBL ProFlora pH-control, JBL Neuhofen, Germany). The growth phase lasted for 14 days, then the biomass was centrifuged (2500 × *g*, 25 min) and approximately half of it harvested, while the remaining half was washed twice with deionized water, suspended in stress phase medium, and exposed for another 14 days. Growth was monitored by spectrophotometric measurement of the optical density (OD) of a subsample at 600 nm. The growth phase reactors were inoculated with the alga at an OD of 0.05. Cell harvest started with a centrifugation at 3000 × *g* for 10 min at 4 °C and the pellet was immediately frozen at − 80 °C. Remaining cells in the supernatant were harvested by vacuum filtration with 125-mm glass microfiber filters (Whatman, Grade GF/B, pore size 1 μm, GE Healthcare, UK) on a suction strainer. Prior extraction for analytics, the material was lyophilized for 48 h and afterwards the total dry mass was weighed.

### Light and transmission electron microscopy

Morphology, cell sizes (*n* = 36), and life cycle were observed by light microscopy (LM) with an Nikon Eclipse 80i LM, Plan Apo VC 100 × 1.40 objective and a DS-5 M camera (Nikon Instruments, Netherlands), either with differential interference contrast or at chlorophyll autofluorescence mode (filter emission = 600 LP, excitation = 480/40). For TEM, 3-week-old cultures were fixed with a standard chemical fixation protocol modified for green algae (2.5% glutaraldehyde, 1% OsO_4_ in 20 mM caccodylate buffer, pH = 7.0) according to Holzinger et al. (2009). Specimens were then dehydrated in increasing ethanol concentrations, transferred to modified Spurr’s resin, and polymerized for 8 h at 80 °C. For observation at the TEM, ultrathin sections were prepared with an ultramicrotome (Reichert Ultracut, Leica Microsystems, Austria), counterstained with uranyl acetate and Reynold’s lead citrate, and examined using a Zeiss LIBRA 120 transmission electron microscope (Carl Zeiss AG, Germany) at 80 kV. Images were captured with a TRS 2 k SSCCD camera (Albert Tröndle Restlichtverstarker Systeme, Moorenweis, Germany) and further processed using Adobe Photoshop software (Adobe Systems Inc., USA).

### DNA extraction, PCR, and sequencing

Total genomic DNA was extracted from strain SAG 2627 using DNeasy Plant Mini Kit (Qiagen, Germany) as described in Procházková et al. (2018). The 18S small subunit ribosomal RNA gene (18S rDNA), internal transcribed spacer region 2 (ITS2 rDNA), and ribulose-1,5-bisphosphate carboxylase/oxygenase large subunit (*rbc*L) marker regions were amplified from DNA isolates by polymerase chain reaction (PCR) using existing primers: primers NS1 (White et al. 1990) and 18L (Hamby et al. 1988), AL1500af (Helms et al. 2001) and LR3 (Vilgalys and Hester 1990), and snow-F0 and snow-R2 (Matsuzaki et al. 2015), respectively. Amplification reactions were described in Procházková et al. (2018). PCR products were purified and sequenced using an Applied Biosystems automated sequencer (ABI 3730xl) at Macrogene Europe (Amsterdam, Netherlands). The obtained sequences were submitted to NCBI Nucleotide sequence database (accession numbers: 18S rDNA + ITS1 + 5.8S rDNA + ITS2 rDNA + partial 26S rDNA: MW866482, *rbc*L: MW854800).

### Phylogenetic analyses

Two different alignments were constructed for the phylogenetic analyses, based on the 18S rRNA and *rbc*L gene sequences. The sequences were selected according to Neustupa et al. (2011) and Barcytė et al. (2017) to encompass all trebouxiophycean lineages. The 18S rDNA alignment (with exclusion of introns) contained 72 sequences (1628 bp), the *rbc*L alignment contained 53 sequences (1194 bp), and *Chlamydomonas reinhardtii* and *Chloromonas rosae* were selected as outgroup. The best-fit nucleotide substitution model was estimated by jModeltest 2.0.1 (Posada 2008). Based on the Akaike information criterion, the TIM2 + I + G and GTR + I + G model was selected for 18S rDNA and *rbc*L, respectively. The 18S rDNA and *rbc*L phylogenetic trees were inferred by Bayesian inference (BI) and maximum likelihood (ML) according to Nedbalová et al. (2017), with the minor modification that Markov chain Monte Carlo runs were carried out for three million generations in BI. Bootstrap analyses and Bayesian posterior probabilities were performed as described by Nedbalová et al. (2017).

### N-glycan extraction and analysis

The N-glycans were isolated by a combination of pepsin digestion, cation exchange–based capturing of peptides and glycopeptides, digestion with peptide:N-glycosidase A (Europa Bioscience Ltd, Cambridge, UK), and repeated cation exchange and polishing by reversed phase solid-phase extraction as described before (Mócsai et al. 2019). Permethylation of native, unreduced N-glycans was carried out in anhydrous dimethyl-sulphoxide in the presence of solid NaOH and a surplus of methyl-iodide following the classical method for carbohydrate permethylation (Ciucanu and Kerek 1984). The released underivatized N-glycans as well as the permethylated N-glycans were subjected to MALDI-TOF mass spectrometry (MS) analysis with dihydroxybenzoic acid as matrix on an Autoflex instrument (Bruker Daltonics, Germany) in the positive ion reflectron mode. The resulted spectra were evaluated with Bruker Daltonics’ flexAnalysis software.

### Fatty acid and pigment analysis

Fatty acids were derivatized, identified, and quantified by gas chromatography and flame ionization detection (Thermo Trace 1300 GC, Thermo Fischer Scientific) according to Remias et al. (2020). Carotenoids, chlorophylls, and alpha-tocopherol were extracted with methyl *tert*-butyl ether and quantified by HPLC with a C30 Carotenoids column (YMC, Willich, Germany) and a diode array detector (Agilent Technologies, Germany) at 450 nm, according to Procházková et al. (2019). The quantitative results were performed in triplicate and the standard derivation (SD) was calculated. Reactor biomass yield (productivity) of a compound class was expressed in dry mass produced per day and liter reactor volume [mg day^−1^ L^−1^], neglecting the minor starting biomass of the inoculum. The yields of the stress phase were calculated in the same manner, however by subtracting the starting biomass and respectively the compound dry weights which were already present in the reactors at the start of the stress phase.

## Results

### Habitat

**Fig. 1:**
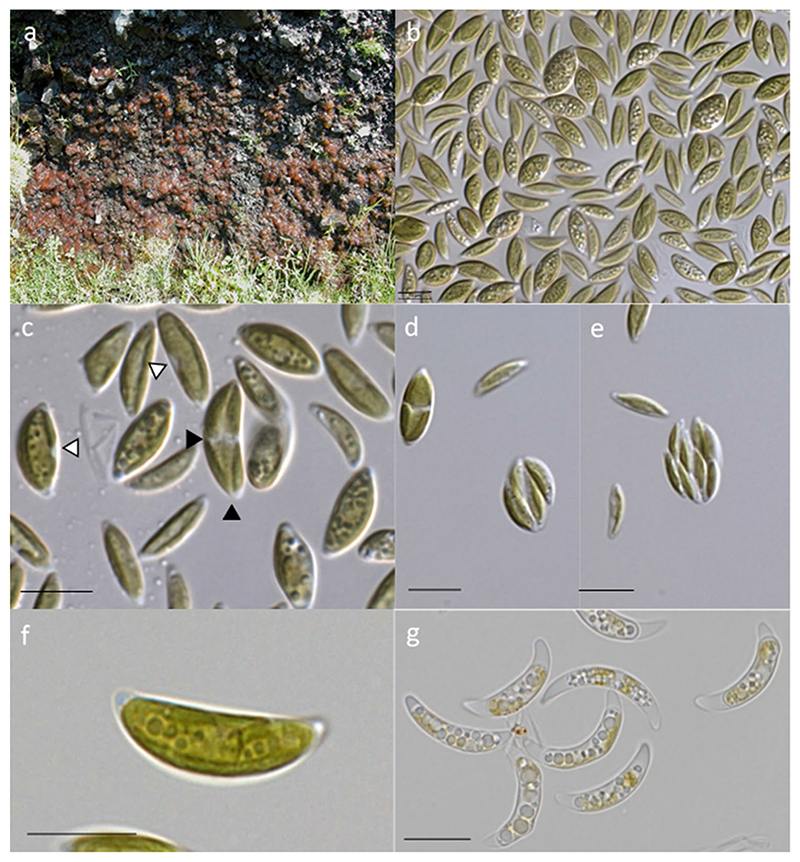
Habitat and light microscopy of *Thorsmoerkia curvula* gen. et sp. nov. SAG 2627 of cells kept in Synthetic Freshwater Medium. **a** Type locality at Iceland, a gravel wall wetted by dripping water and dominated by reddish mucilage caused by filamentous cyanobacteria. **b** Group of cells **c** older cells showing characteristic incisions of the chloroplast (white arrowheads). Prior cell division, the chloroplast shows incisions at the equatorial and vertical plane (black arrowheads). **d** Four cells after division, **e** eight cells after division. **f** Detail view of a grown cell, **g** prominently curved and vacuolated cells in stress (nitrogen and phosphate depleted) medium. Scale: 10 μm

The collection site was made of a steep gravel wall which was wet from dripping water from above. Photoautotrophic mats of mucilaginous consistency and brownish-orange color were dominating (Fig. 1a), and the pigmentation was obviously caused by abundant filamentous cyanobacteria (cf. Nostocales, data not shown). The species selected for this study was one of several green microalgae which were light microscopically observed to be present in this species rich community.

### Cell morphology and ultrastructure

LM of the strain SAG 2627 revealed a characteristic, elongate, more or less crescent cell morphology with conical poles for all observed stages (Fig. 1b). Close to each cell pole, a small vacuole was located in the cytoplasm (Fig. 1f), whereas larger vacuoles may appear more central (Fig. 1g). Cells had a channel-shaped to coat-like parietal chloroplast without visible pyrenoid and with few incisions at older, larger cells. Flagellate stages or sexual reproduction was not observed. Mature cells possess frequently a chloroplast emargination at the equatorial and vertical planes (Fig. 1c). Prior cell division, a sectioning of the chloroplast into four equal pieces was observed (Fig. 1c). After cytogenesis, the autosporangia consisted either of four or eight smaller daughter cells of the same shape like the older cells (Fig. 1d and e). The average cell sizes were 14.4 ± 2.2 μm length and 5.3 ± 1.4 μm width. Nutrient-starving cells at the end of the stress phase were stronger curved and exhibited a significant cytosolic vacuolization (Fig. 1g). When cells were kept at a lowered temperature (8 °C) for a prolonged period, several became larger and more roundish.

**Fig. 2:**
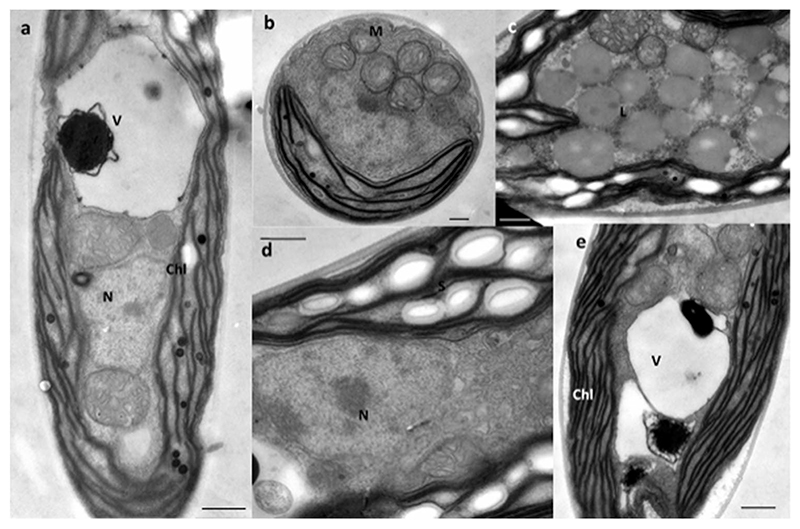
Transmission electron micrographs of *Thorsmoerkia curvula* gen. et sp. nov. SAG 2627 **a** Longitudinal median section illustrating a large vacuole with an osmiophilic body inside. **b** Cross-section in a central position showing the nucleus, the parietal chloroplast and many mitochondria indicating a high metabolic activity. **c** Older cell with cytoplasmic lipid bodies. **d** Parietal chloroplast containing many starch grains, but no pyrenoid, the nucleus is in a central position surrounded by dense cytoplasm with ER. **e** Chloroplast with prominent thylakoid stacks. Abbreviations: V vacuole, M mitochondrion, N nucleus, S starch grain, chl chloroplast. Scale bars a, c–e) 0.5 μm, b) 0.25 μm

By means of TEM (Fig. 2a–e), the ultrastructure of the cells was examined, which corroborated the LM observations. The nucleus with a distinct nucleolus was in a slightly acentric position (Fig. 2a, b, d) surrounded by numerous mitochondria (Fig. 2a, b). The chloroplast had a regular appearance with parallel-arranged thylakoids and occasional plastoglobules (Fig. 2a, b, e). Starch grains were located inside the chloroplasts and accumulated between thylakoid membranes but were not associated with a pyrenoid. In the central part of the cells, accumulations of lipid bodies were visible (Fig. 2b).

### Molecular phylogeny

**Fig. 3:**
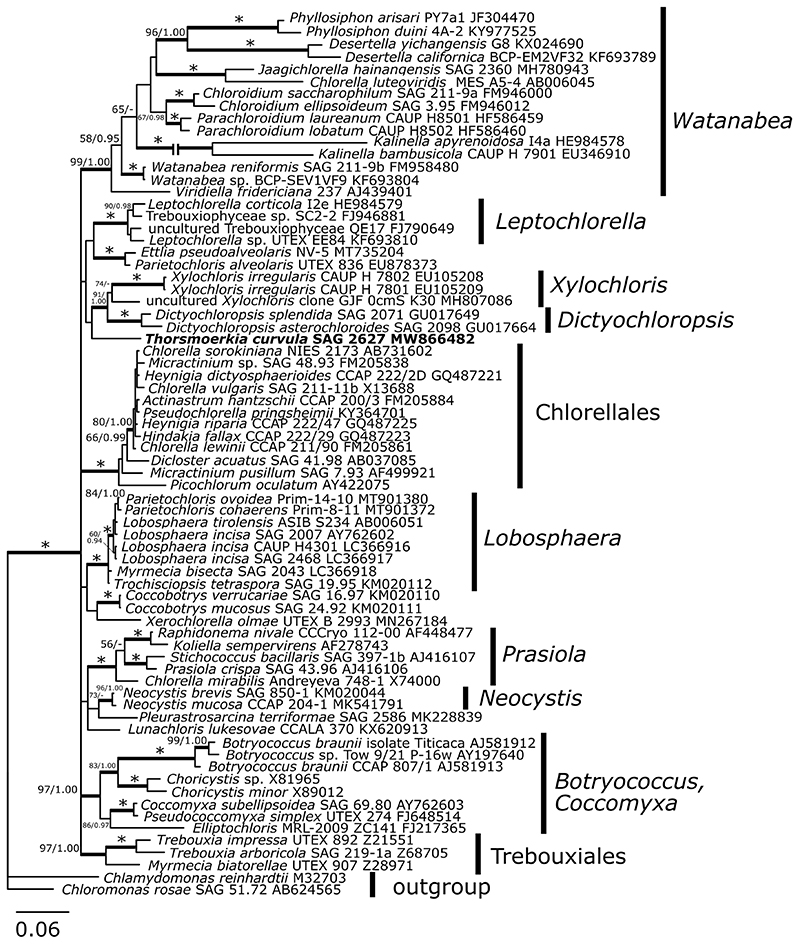
18S ribosomal RNA gene-based Bayesian phylogenetic tree including *Thorsmoerkia curvula* SAG 2627 (in bold) and other Trebouxiophyceae. Bootstrap values from maximum likelihood analysis (≥ 50%) and posterior probabilities (≥ 0.95) are shown. Full statistical support (1.00/100) is marked with an asterisk. Thick branches represent nodes receiving the highest posterior probability support (1.00). Accession numbers, strain, or field sample codes are indicated after each species name

**Fig. 4:**
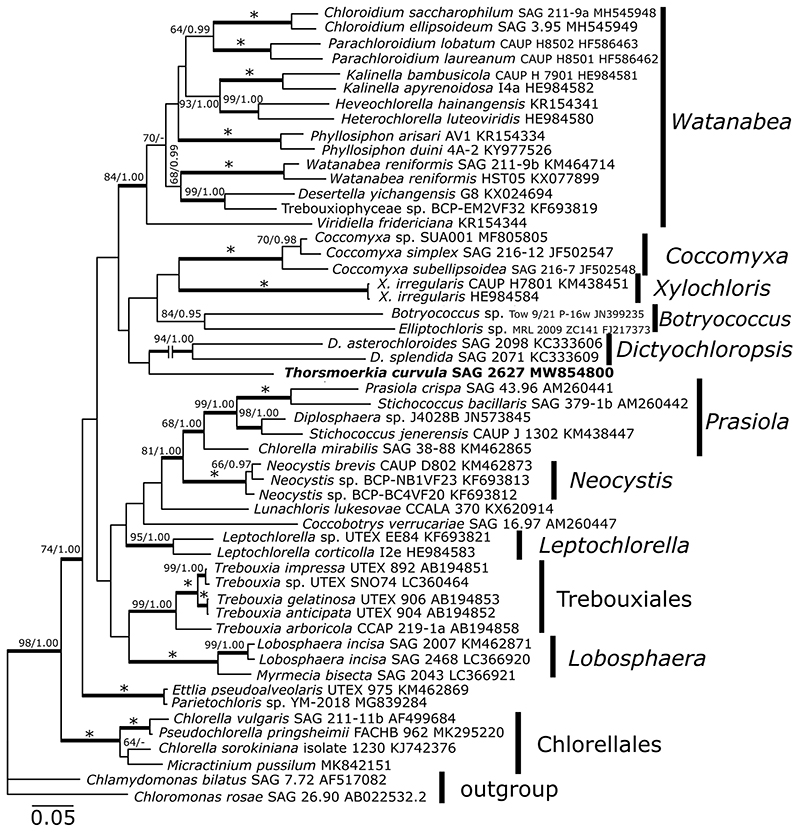
*rbc*L gene–based Bayesian phylogenetic tree including *Thorsmoerkia curvula* SAG 2627 (in bold) and other Trebouxiophy- ceae. Bootstrap values from maximum likelihood analysis (≥50%) and posterior probabilities (≥ 0.95) are shown. Full statistical support (1.00/100) is marked with an asterisk. Thick branches represent nodes receiving the highest posterior probability support (1.00). *D*. = *Dictyochloropsis*, *X*. = *Xylochloris*. Accession numbers, strain or field sample codes are indicated after each species name

The 18S rDNA sequences of strain SAG 2627 contained a single intron (588 bp long), inserted between positions 1143 and 1144 (positions numbers in relation to the reference sequence of *Chlamydomonas reinhardtii*, GenBank: JN903978). In BLAST searches against the nr database at NCBI, the 18S rDNA and *rbc*L sequences of the alga were most similar to sequences of the Trebouxiophyceae; it showed 96% sequence similarity in 18S rDNA to several taxa such as *Myrmecia bisecta* SAG 2043 (LC366918), *Lunachloris lukesovae* CCALA 370 (KX620913), and *Coccobotrys mucosus* SAG 24.92 (KM020111). The *rbc*L comparisons revealed a relatively low similarity of 86% for the closest match, such as *Trebouxia impressa* UTEX 892 (AB194851) and *Ettlia pseudoalveolaris* UTEX 975 (KM462869). In 18S rDNA (Fig. 3) and *rbc*L (Fig. 4) analysis, strongly supported lineages (i.e., BI > 0.94, ML > 79%; Škaloud and Peksa 2010) corresponding to the genera *Watanabea*, *Leptochlorella*, *Xylochloris*, *Dictyochloropsis*, *Dictyosphaerium* belonging to the order Chlorellales, *Lobosphaera*, *Prasiola*, *Neocystis*, *Botryococcus*/*Coccomyxa* from the order Trebouxiales as defined by Neustupa et al. (2011) and Barcytė et al. (2017) were recovered. The strain of this study was placed in a distinct sister position to *Dictyochloropsis* and *Xylochloris*-clades in ML and BI, but neither topology was statistically supported.

For the ITS1 spacer region, there were no blast hits. Regarding the 5.8S-ITS2 spacer region, the closest GenBank relatives of the studied strain comprised several Trebouxiophyceaen taxa and had quite low identities, e.g., *Pseudochlorella pyrenoidosa* SAG 18.95 (AM422986; 84% identic, 296 bp of 353 bp), uncultured Chlorophyta clone ALCG12 (JX435387.1; 85% identic, 274/324), clone PA2009A25 (HQ191320.1; 85% identic, 256/298), or *Dictyochloropsis splendida* SAG 2071 (GU017649.1; 81% identic, 251/309).

#### Thorsmoerkia Remias & Procházková, gen. nov

DESCRIPTION: Vegetative cells solitary and uninucleate. Cells have an oblong to crescent shape with conical poles. The chloroplast is parietal. Reproduction takes place by means of 4 or 8 autospores of the same shape like the mother cell. Flagellate stages or sexual reproduction unknown. The genus differs from other members of Trebouxiophyceae by the 18S rDNA and *rbc*L sequences. The generic name reflects the distinct curved cell shape.

ETYMOLOGY: The generic name reflects the geographic region Þórsmörk (“Thor’s Forest”) at Iceland, from where the alga was isolated.

TYPE SPECIES OF THE GENUS: *Thorsmoerkia curvula* Remias & Procházková.

#### Thorsmoerkia curvula Remias & Procházková, sp. nov. Figure 1 a-g

DESCRIPTION: Vegetative cells solitary with a crescent cell morphology and with conical poles. Single nucleus located non-centrally. The dimensions of vegetative cells are 9.1 – 17.4 × 2.7 – 9.3 μm. Single channel-shaped to coat-like parietal chloroplast. Reproduction by means of 4 or 8 autospores 10.2 ± 0.9 μm long and 3.3 ± 0.6 μm wide. Stressed (nutrient depleted) cells contain higher numbers of cytosolic lipid bodies. Flagellate stages or sexual reproduction was not observed. The species differs from other species in 18S rDNA, ITS2 and *rbc*L sequences. DNA sequences available for the type strain: nuclear 18S rDNA-ITS1-5.8S-ITS2-26S rDNA (MW866482) and plastid *rbc*L (MW854800).

HABITAT: volcanic wet gravel, semi-aerophytic

TYPE LOCALITY: N63°42.144, W19°40.194, 157 m a.s.l., Þórsmörk, Iceland.

HOLOTYPE HERE DESIGNATED: preserved specimen WP156 fixed for TEM embedded material of strain SAG 2627 at the Department of Botany, University of Innsbruck, Austria.

STRAIN EXAMINED: This strain was deposited at The Culture Collection of Algae at Göttingen (SAG), Germany, as SAG 2627.

ETYMOLOGY: The species epithet *curvulaʾ* reflects the shape of cells that are slightly curved.

### Protein N-glycosylation

**Fig. 5:**
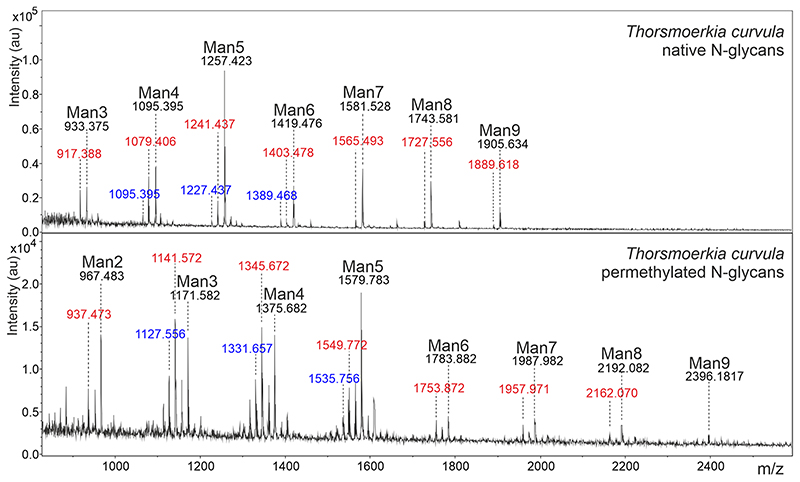
MALDI-TOF MS spectra of N-glycans of *Thorsmoerkia curvula* SAG 2627. The upper panel shows the native, unreduced glycans, the lower panel presents the result obtained after permethylation. The conventional oligomannosidic glycans are labelled as Man2 to Man9. Another glycan series was observed to contain one deoxyhexose and one to eight hexoses (red labels). A single pentose was found on smaller oligomannosidic glycans (blue labels). N-glycans with both a pentose and a deoxyhexose residue were not detected. Unlabeled peaks are undesired side-products of the derivatization process. Note that MALDI-TOF MS does not reliably reflect the true ratios of N-glycans as signals in the higher mass range are disproportionally weaker

MALDI-TOF MS of the unreduced N-glycans of *T. curvula* SAG 2627 presented a consistent series of masses revealing oligomannosidic N-glycans consisting of two N-acetylhexosamines and 2 to 9 hexose residues, presumably mannoses (Fig. 5). Another series of N-glycans with 1–8 hexoses featured a mass increment of m/z = 146 that could derive either from a deoxyhexose or a pentose in combination with a methyl group. This ambiguity was resolved by permethylation of the sample, which revealed the occurrence of a deoxyhexose, hitherto unprecedented in Trebouxiophyceae. There were also traces of a single pentose containing glycan series on smaller glycans. N-glycans with both a pentose and a deoxyhexose residue were, however, not detected. Noteworthy, the N-glycans lacked methylation.

### Biomass and FA yields

The biotechnological productivity was tested under four different conditions, i.e., growth and stress phase, each performed either without or with CO_2_ supplementation. Highest biomass yield was recorded during “growth + CO_2_” with 53.50 ± 1.86 mg DM L^−1^ day^−1^, followed by “growth” at 35.31 ± 0.06 mg DM L^−1^ day^−1^. The numbers were lower during stress without (14.57 ± 2.29) and even lower with CO_2_ (1.75 ± 0.51 mg DM L^−1^ day^−1^), respectively. In a similar manner, the yield of FAs was best for “growth + CO_2_” at 23.97 ± 1.39 mg L^−1^ day^−1^, followed by “growth” at 9.64 ± 0.61, and the lowermost values for the stress phases with 4.72 ± 0.44 and 0.89 ± 0.26 mg DM L^−1^ day^−1^. Table 1 shows the productivity divided into un-, mono-, and polyunsaturated FAs and Table 2 gives the relative proportions of the main FAs in all four treatments. The highest eicosapentaenoic acid (EPA; C20:5) content was at the end of the “growth” phase (8.47% of all FAs), likewise the EPA-yield was 0.81 mg L^−1^ day^−1^.

### Pigment and α-tocopherol yields

The main pigments of *T. curvula* SAG 2627 were chlorophylls and lutein; minor amounts of violaxanthin, neoxanthin, and alpha-/beta-carotene were detected as well (data not shown). In general, the “stress” treatments did not result in a color change of the cells from green to reddish, accordingly, no secondary carotenoids were detected. Total carotenoid yield was best after “growth” with 2.94 ± 0.11 mg L^−1^ day^−1^, followed by “growth + CO_2_” at 2.08 ± 0.06 mg L^−1^ day^−1^. The cells exposed to stress assays showed hardly carotenoid production (0.07 ± 0.01 mg L^−1^ day^−1^), or even a decline without additional CO_2_ (–0.03 ± 0.09 mg L^−1^ day^−1^). α-Tocopherol yield behaved in a different manner with similar values for growth with and without CO_2_ (0.17 ± 0.03 or 0.19 ± 0.01 mg L^−1^ day^−1^, respectively) and a reduction to about the half of the initial values during stress (0.09 ± 0.05 and 0.09 ± 0.02 mg L^−1^ day^−1^).

## Discussion

The collection site in a river valley was apparently a suitable habitat for semi-terrestrial microalgae and cyanobacteria. On the one hand, possibly due to modest but constant drip water supply from above at the gravelly terrace wall of Markarfljót river, which served as a substrate. On the other hand, this steep cliff was less affected by intense grazing common in large parts of Iceland, likely resulting in less nitrification and a local higher diversity of microalgae and vascular plants (own observations, data not shown). We did not test the water chemistry at the sampling site, but the alga grew better in SFM with an initial pH of 7 instead of 6 (data not shown).

The characteristic, more or less crescent outer cell shape of *Thorsmoerkia curvula*, is not unique; several green microalgae similar in size and morphology from likewise habitats are known, within and outside the Trebouxiophyceae. Krienitz et al. (2011) already showed the polyphyletic origin of crescent-shaped green algae by molecular means, and recognized them in three clades of the Trebouxiophyceae and in ten clades of Selenastraceae. In the latter, the new species resembled the terrestrial *Monoraphidium pusillum* (Ettl and Gärtner 2013). It also looks similar to members of the genus *Ankistrodesmus* (both are Chlorophyceae *s. str.*); interestingly, George et al. (2014) reported that the cell shape, size, and tendency to form clusters may change depending on the medium composition. In contrast to the species of this study, the members of the *Dictyochloropsis* clade, which seems to be phylogenetically closest (but without statistic support), have a prominent reticulate chloroplast and no crescent cell morphology (Geitler 1966). *Dictyochloropsis* also makes autosporangia like the here investigated strain but differs morphologically by a spherical to ellipsoidal cell shape (Škaloud et al. 2016). Thus, and because of the distinct phylogenetic position, we propose the new gen et. sp. *T. curvula*. Sexual reproduction was not observed for *T. curvula*. On the one hand, occasionally irregularly shaped stages were observed, suggesting the process of syngamy. Accordingly, the formation of gametes, fertilization, and development of planozygotes has been occasionally observed for trebouxiophycean algae (Škaloud et al. 2016). Fučíková et al. (2015) summarized reports including direct observation of syngamy, the formation of a zygote, or stages presumed to be the result of syngamy in a limited number of species in the orders Chlorellales, *Elliptochloris* clade, *Prasiola* clade, and order Trebouxiales. Moreover, meiotic genes were found in all examined genomes and transcriptomes of this group, even in species presumed to be asexual (Fučíková et al. 2015). Similarly, sex-related genes underlying sexual reproduction in *Chlorella sorokiniana* were found (Hovde et al. 2018) suggesting that cryptic sex is prolific throughout the Trebouxiophyceae.

The 18S rDNA and *rbc*L phylogenetic trees of Trebouxiophyceae presented in this study are congruent with phylogenies previously presented for this class (e.g., Barcytė et al. (2017), still, specifying the exact position of the genus *Thorsmoerkia* may require acquiring sequence data from potentially closer relatives. This is congruent with the findings that the ITS1-5.8S-ITS2 rDNA region of *T. curvula* was highly divergent compared to other trebouxiophycean taxa explored so far. Nevertheless, according to the 18S rDNA and *rbc*L gene–based phylogenies (Figs. 3 and 4), *T. curvula* is not closely related to any of the morphologically similar trebouxiophycean taxa, including young cells of “E-form” of *Watanabea reni-formis* (Hovde et al. 2018) or *Neocystis mucosa* (Krienitz et al. 2011). In the last years, several new lineages of trebouxiophycean algae have been discovered including the description of new genera, suggesting that the diversity of this group is still underexplored (Neustupa et al 2013; Song et al 2015; Vančurová et al. 2015; Ulrich and Röske 2018; Darienko and Pröschold 2019). This goes along with a recent metagenomic study showing that the most diverse trebouxiophycean communities are found in freshwater and soil environments (Metz et al. 2019). Like in the present study, integrative taxonomic approaches combining morphological and molecular traits will improve the knowledge about biodiversity in the diverse class of Trebouxiophyceae (e.g., Muggia et al. 2018).

Taxonomy of microalgae has been a constant challenge; therefore, a robust and reliable biomarker is a welcomed asset in distinguishing different species. Recently, N-glycans have been suggested to be a promising candidate to achieve this goal as they facilitated to differentiate even between closely related species of Chlorellaceae (Mócsai et al. 2020a). This method has not yet been applied to a broad spectrum of microalgae, but still presents an opportunity to utilize when it comes to new species of Trebouxiophyceae, potentially complementing molecular data. Observing the full series of oligomannosidic N-glycans in *T. curvula* suggests that the organism has all the enzymes involved to complete the conventional lipid-linked oligosaccharide precursor. Although this is a conserved feature and all higher plants, yeasts, and even vertebrates possess this common biochemical pathway, it was recently shown that not all green algae are processing their N-glycans the conventional way. For example, *Chlamydomonas reinhardtii* was synthesizing a linear Glc_3_Man_5_GlcNAc_2_ precursor (Lucas et al. 2018), unprecedented anywhere else in eukaryotic kingdoms. The most important observation in *T. curvula* is that a deoxyhexose was not present on the same glycan as a pentose, as if they would both sterically hinder the further maturation of the glycan. This might suggest that the deoxyhexose and/or the pentose present in *T. curvula* N-glycans structurally differ from the plant-specific linkages of β1,2-xylose to β-mannose and α1,3-fucose to the first GlcNAc residue. Another important feature of *T. curvula* protein N-glycosylation is that the N-glycans lacked methylation, which is often observed in other Trebouxiophyceae, e.g., *Chlorella* species (Mócsai et al. 2019, 2020a, b). The biological function of these N-glycans remains an open question, but involvement in cell–cell interaction is possible. In summary, the novel species from Iceland exhibits a hitherto unseen N-glycosylation pattern within the Trebouxiophyceae, which supports the hypothesis that taxa distinguish themselves by subtle features of their N-glycosylation. Eventually, this chemotaxonomic hypothesis will be assessed only when more microalgae will be subjected to N-glycosylation experiments. Still, the fact that microalgae have diverse N-glycan patterns reflect that their biochemical pathways of protein N-glycosylation are much less conserved than those observed in higher plants. This opens up the possibility of a new method for microalgae classification.

Aerated column reactors with pH-regulated, episodically CO_2_ supplementation proved to be ideal to effectively generate biomass of *T. curvula*. The present study did not investigate the temperature optima of the newly described alga, but a temperature range between approximately 20 to 25 °C was most suitable for growth. The alga exhibited a fatty acid content of almost 45% per dry mass already during the growth phase. Likewise, the EPA content peaked at the same conditions. Consequently, the application of a stress protocol with depleted medium is not feasible for this species, considering also that, unlike other green algae, an increased production of carotenoids was not observed. Maltsev et al. (2018) showed that the qualitative profile of fatty acids can be influenced by modulating the phosphate and nitrogen contents of the medium, but their trebouxiophycean soil alga *Parietochloris grandis* reached only about 20% FAs per dry weight during growth phase. A bioprospecting study of microalgae by Steinrücken et al. (2017) showed, like in this study, that species from high latitudes are suitable for biotechnological applications, and diatoms of marine origin outcompeted *T. curvula* in production of valuable PUFAs like EPA. Besides lipids, Trebouxiophyceae are a well-known source for mycosporine-like amino acids (MAAs) effectively absorbing UV irradiation or for compatible solutes such as polyols (Hotter et al. 2018). MAAs were detected in *Thorsmoerkia curvula* (M. Ganzera, Institute of Pharmacy, University of Innsbruck, pers. comm.) but not further characterized yet. In a biotechnological point of view, this alga is a primarily a source of valuable lipids due to the presence of high amounts of unsaturated fatty acids, to be cultivated under modest light conditions in freshwater with a convenient one-phase approach.

## Supplementary Material

**Supplementary Information** The online version contains supplementary material available at https://doi.org/10.1007/s10811-021-02577-y.

Supplementary material

## Figures and Tables

**Table 1:** Contents of the three FA classes in *Thorsmoerkia curvula* SAG 2627 [in % of DM, ± standard deviation, *n* = 3] for all 4 cultivations. Abbreviations: *gr.*, growth; *MUFA*, monounsaturated fatty acids; *PUFA*, polyunsaturated fatty acids; *SFA*, saturated fatty acids; *str.*, stress

	Growth	Stress	gr. + CO_2_	str. + CO_2_
SFA	5.47 ± 0.3	10.03 ± 0.3	6.88 ± 0.2	7.42 ± 0.2
MUFA	10.12 ± 1.0	37.31 ± 1.4	26.25 ± 0.8	30.08 ± 0.2
PUFA	11.72 ± 0.4	12.72 ± 0.0	11.68 ± 0.3	13.21 ± 0.2
Sum	27.31	60.06	44.81	50.71

**Table 2:** Percentage of the main FA size classes at all 4 cultivations of *Thorsmoerkia curvul* *a* SAG 2627. Abbreviations: *gr.*, growth phase; *str.*, stress phase

	Growth	Stress	gr. + CO_2_	str. + CO_2_
C12:0	0.16	0.05	0.06	0.06
C14:0	2.47	2.75	4	3.78
C16:0	14.23	10.4	8.45	8.01
C16:1	0.97	0.52	0.4	0.35
C18:0	2.25	2.91	1.98	1.93
C18:1	36	61.58	58.17	58.98
C18:2	10.29	6.67	8.66	9.68
C18:3	24.25	10.49	14.16	12.96
C20:0	0.12	0.13	0.12	0.12
C20:5	8.47	4.04	3.25	3.4
C22:0	0.26	0.18	0.2	0.24
C24:0	0.55	0.28	0.54	0.49
Total	100	100	100	100
