# Supplementary material for: Thorsmoerkia curvula gen. et spec. nov. (Trebouxiophyceae, Chlorophyta), a semi-terrestrial microalga from Iceland exhibits high levels of unsaturated fatty acids
Source: J Appl Phycol. Author manuscript; Available in PMC 2022 Mar 17. (PMC7612509; doi:10.1007/s10811-021-02577-y)
Supplement: Supplementary material [file EMS143828-supplement-Supplementary_material.pdf]

Supplementary Material

Thorsmoerkia curvula gen. et spec. nov. (Trebouxiophyceae, Chlorophyta), a semi-terrestrial microalga from Iceland exhibits high levels of unsaturated fatty acids

Journal of Applied Phycology

Cecilia Nicoletti, Lenka Procházková, Linda Nedbalová, Réka Mócsai, Friedrich Altmann, Andreas Holzinger, Daniel Remias

Corresponding author: Daniel Remias Daniel.Remias@fh-wels.at, University of Applied Sciences Upper Austria, Austria

Supplemental Table 1 Composition of "enhanced Synthetic Freshwater Medium" (eSFM). Prepare the stock solutions first and then add the indicated volume of each stock solution 1 to 5 and 7 to 950 mL of deionized water (dH_2O). Then adjust the pH with 4M HCl to 7.0. Bring to a final volume of 1 liter and autoclave (121°C, 15 min). Stock solution 6 (vitamins) should have a pH of 7 and be stored in the fridge. Add it to the autoclaved medium through a sterile membrane filter. The Trace Metals stocks should be stored frozen in plastic bottles. The stress medium used in this study lacking nitrogen and phosphate ("-N -P SFM") was prepared by omitting stock 4 and 6 and substituting stock 2 of eSFM with $CaCl_2 \cdot 2\ H_2O$ (3.09 g / 100 mL dH_2O), adding 2 mL to prepare 1 liter of final medium

Stock	Component	Stock solution	added for 1 L medium
**1.**	HEPES puffer	238.10 g / L dH_2O	1 mL
**2.**	$Ca(NO_3)_2 \cdot 4\ H_2O$	100.00 g / L dH_2O	1 mL
**3.**	$MgSO_4 \cdot 7\ H_2O$	20.00 g / L dH_2O	2.5 mL
	$K_2HPO_4 \cdot 3\ H_2O$	5.00 g / L dH_2O	
**4.**	$NaNO_3$	50.00 g / L dH_2O	1.2 mL
	Na_2CO_3	32.00 g / L dH_2O	
**5.**	H_3BO_3	1.00 g / L dH_2O	1 mL
**6.**	***Vitamin Solution:***		1 mL
	Vitamin B_{12}	0.20 mg / L dH_2O	
	Biotin (Vitamin H)	1.00 mg / L dH_2O	
	Thiamine-HCl (Vitamin B_1)	100.00 mg / L dH_2O	
	Niacinamide (Vitamin B_3)	0.10 mg / L dH_2O	
**7.**	***Trace Metals:***		1 mL
**7.1.**	Preparation of Trace Metal Solution:		
	$Na_2EDTA \cdot 2\ H_2O$: 4.36 g		
	$FeCl_3 \cdot 6\ H_2O$: 3.15 g		
	Dissolve 7.1. in 1 l dH_2O, then add 1 mL of Primary Trace Metal solutions each:		
**7.2.**	***Primary Trace Metals:***		
**7.2.1.**	K_2CrO_4	0.194 g / 100 mL dH_2O	
**7.2.2.**	$CoCl_2 \cdot 6\ H_2O$	1.00 g / 100 mL dH_2O	
**7.2.3.**	$CuSO_4 \cdot 5\ H_2O$	0.25 g / 100 mL dH_2O	

**7.2.4.**	$MnCl_2 \cdot 4\ H_2O$	18.00 g / 100 mL dH_2O
**7.2.5.**	$Na_2MoO_4 \cdot 2\ H_2O$	1.89 g / 100 mL dH_2O
**7.2.6.**	$NiSO_4 \cdot 6\ H_2O$	0.27 g / 100 mL dH_2O
**7.2.7.**	H_2SeO_3	0.13 g / 100 mL dH_2O
**7.2.8.**	Na_3VO_4	0.184 g / 100 mL dH_2O
**7.2.9.**	$ZnSO_4 \cdot 7\ H_2O$	2.20 g / 100 mL dH_2O

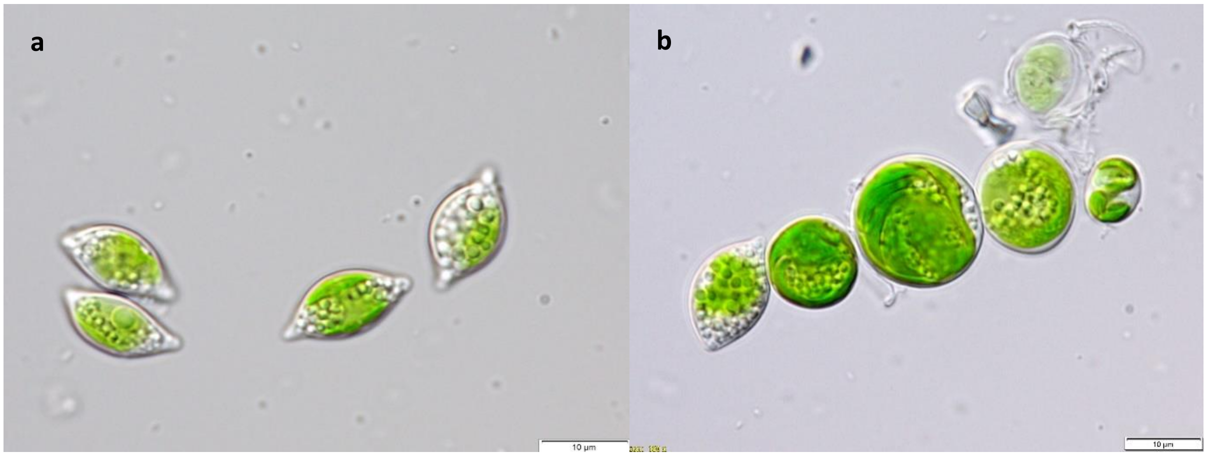

Supplementary Fig. 1 **a** When *Thorsmoerkia curvula* was kept at 8°C and an irradiation of 35 µmol photons $m^{-2}\ s^{-1}$, thicker cells with average sizes 15.1 ± 2.7 µm x 16.5 ± 2.6 µm and size ranges 11.4-21.2 µm x 12.9-21.4 µm appeared. **b** Occasionally, roundish stages without conical cell poles and with altered chloroplast morphology were developed in the depleted medium
